# Telomerase Inhibition by a New Synthetic Derivative of the Aporphine Alkaloid Boldine

**DOI:** 10.3390/ijms19041239

**Published:** 2018-04-19

**Authors:** Sakineh Kazemi Noureini, Mitra Kheirabadi, Fatima Masoumi, Farve Khosrogerdi, Younes Zarei, Cristian Suárez-Rozas, Julio Salas-Norambuena, Bruce Kennedy Cassels

**Affiliations:** 1 Department of Biology, Faculty of Sciences, Hakim Sabzevari University, Sabzevar 9617976487, Iran; masoumifatima@gmail.com (F.M.); fkhosrogerdi@gmail.com (F.K.); Zarei.6171@gmail.com (Y.Z.); 2 Department of Chemistry, Faculty of Sciences, University of Chile, Santiago 1058, Chile; cristiansuarezro@gmail.com (C.S.-R.); julio.salas@umce.cl (J.S.-N.)

**Keywords:** telomerase inhibition, boldine, *N*-benzylsecoboldine, derivative, binding site

## Abstract

Telomerase, the enzyme responsible for cell immortality, is an important target in anti-cancer drug discovery. Boldine, an abundant aporphine alkaloid of *Peumus boldus*, is known to inhibit telomerase at non-toxic concentrations. Cytotoxicity of *N*-benzylsecoboldine hydrochloride (BSB), a synthetic derivative of boldine, was determined using the MTT method in MCF7 and MDA-MB231 cells. Aliquots of cell lysates were incubated with various concentrations of BSB in qTRAP (quantitative telomere repeat amplification protocol)-ligand experiments before substrate elongation by telomerase or amplification by hot-start Taq polymerase. The crystal structure of TERT, the catalytic subunit of telomerase from *Tribolium castaneum*, was used for docking and molecular dynamics analysis. The qTRAP-ligand data gave an IC_50_ value of about 0.17 ± 0.1 µM for BSB, roughly 400 times stronger than boldine, while the LD_50_ in the cytotoxicity assays were 12.5 and 21.88 µM, respectively, in cells treated for 48 h. Although both compounds interacted well with the active site, MD analysis suggests a second binding site with which BSB interacts via two hydrogen bonds, much more strongly than boldine. Theoretical analyses also evaluated the IC_50_ for BSB as submicromolar. BSB, with greater hydrophobicity and flexibility than boldine, represents a promising structure to inhibit telomerase at non-toxic concentrations.

## 1. Introduction

Boldine (1,10-dimethoxy-2,9-dihydroxyaporphine) is an aporphine alkaloid found in several plant species and is the main alkaloid in the bark and a relatively abundant component of the leaves of the boldo tree (*Peumus boldus*) [1] and *Lindera aggregata* [2]. Boldine is known for its health promoting properties that include hepatoprotective, cytoprotective, antipyretic and anti-inflammatory effects [3]. Its traditional use for the treatment of gastrointestinal disorders may be based on its antagonistic effects on 5-HT_3_ receptors [4]. The potent antioxidant effects of boldine guarding nitric oxide against reactive oxygen species have been shown to protect the endothelium, supporting its therapeutic role against hypertension and diabetes mellitus [5,6,7].

Boldine has also been shown to reduce the viability and proliferation of T24 human bladder carcinoma cells by inducing cell cycle arrest at the G2/M-phase and causes cell death by apoptosis in correlation with AKT inactivation and glycogen synthase kinase-3β (GSK-3β) activation [8]. Boldine showed a strong induction of apoptosis in breast cancer cells [9], and also cerebrovascular protective effects against neural apoptosis via inhibition of mitochondrial Bax translocation and cytochrome C release [10].

Our previous studies have shown antiproliferative effects of boldine on several cancer cell lines including HepG2, MCF7 and MDA-MB-231 at non-toxic concentrations, and identified its promising potential in telomerase inhibition [11,12]. Telomerase, as a key target in cancer drug discovery, is believed to be mainly regulated at the transcription level of its catalytic subunit, TERT. Boldine affected regulation of this ribonucleoprotein at various points, although no interaction with telomere sequences was detected for boldine. Therefore, substrate sequestration is not involved in its anti-telomerase effects, but the active telomerase content of the treated cells was reduced dose- and time-dependently through transcriptional down-regulation [12].

**Figure 1:**
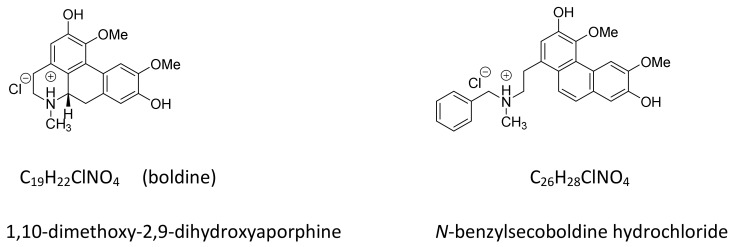
Chemical structures of boldine and *N*-benzylsecoboldine (BSB) hydrochlorides.

On the other hand, direct incubation of cancer cell extracts with boldine also resulted in enzyme inhibition verified by the quantitative TRAP-ligand assay. Boldine may interact directly with the enzyme, but where and how it may bind to the enzyme are still unknown. Here, we studied the mode of interaction of boldine and its derivative *N*-benzylsecoboldine hydrochloride (Figure 1) with TERT, the catalytic subunit of telomerase, using computational simulations and inhibition potential. 

## 2. Results and Discussion

### 2.1. Cytotoxicity of Boldine Derivatives

The MTT test showed that *N*-benzylsecoboldine (BSB) hydrochloride has LD_50_ = 16.25 and 21.88 µM against the MCF7 and MDA-MB-231 lines, respectively. Also, microscopic observation showed that the survival percentage of cells in the presence of BSB was significant and was dose-dependently reduced. 

### 2.2. N-Benzylsecoboldine (BSB) Inhibited Telomerase at Nanomolar Concentrations in a Direct Interaction

**Figure 2:**
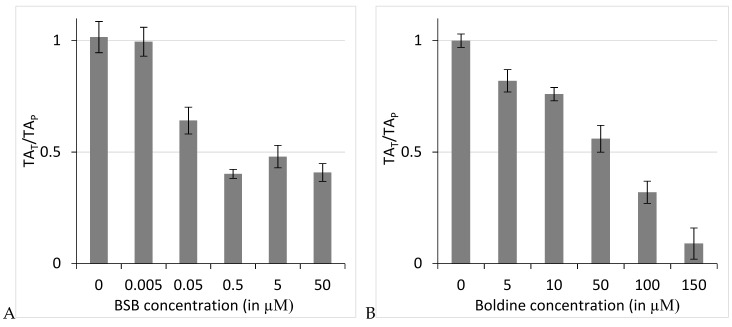
Relative telomerase activity of q-TRAP-ligand reactions when treated with *N*-benzylsecoboldine (BSB) (**A**) or boldine (**B**) before telomere elongation to q-TRAP-ligand reactions when treated after telomere elongation. The mean value ± SEM is presented.

Among the boldine derivatives tested, BSB, by far the most cytotoxic compound against both cell lines, was further studied for its anti-telomerase potential. Figure 2 represents the ratio of telomerase measurements when BSB (A) or boldine (B) was added before telomere elongation (TA_T_) to telomerase activity measurements when the compound was added before the amplification step (TA_P_) obtained from q-TRAP-ligand assay. Telomerase activity was reduced to ≤50% of untreated samples, at BSB concentrations ≤0.5 µM, although not completely suppressed at higher doses. The inhibitory effect of boldine on telomerase activity was dose-dependent so that in the presence of 150 µM boldine telomerase activity fell to around 10% of the control reaction. However, the technique calculated 0.17 ± 0.1 and 68 ± 2.5 µM as IC_50_ values for BSB and boldine, respectively. The differences between untreated control samples and the treated samples are significant, as analyzed using one-way ANOVA with the post-hoc Tukey HSD test (*p* < 0.01). The q-TRAP-ligand assay showed that BSB strongly inhibits telomerase in a direct interaction (Figure 2). This shows a significantly greater inhibition potential of BSB when compared with the parent aporphine boldine. We then investigated whether and how BSB might interact with the enzyme.

In our q-TRAP-ligand experiments BSB did not reduce the activity of Taq polymerase, and no significant decrease was seen in TRAP products in set B; BSB does not interfere with Taq activity, nor with the protein or substrate binding. Therefore it seems likely that this derivative binds to the telomerase at a different site than the general domains existing in DNA polymerase enzymes. If it interrupts the reverse transcription activity of the telomerase, it seems possible that it might interact with the RT domain.

### 2.3. Binding Site of BSB on TERT

The active site of the enzyme is usually the most important target site in ligand binding studies. In our computational studies on telomerase interactions with boldine and BSB, we started with the active site of TERT, but we noticed that some extra binding site(s) might exist. Therefore, we looked for binding site(s) on two levels: focused and blind docking.

#### 2.3.1. Docking Studies of Boldine and Derivatives to Telomerase

Among animal species, telomerase enzymes have highly conserved motifs A and C in the RT palm region and have signature conserved amino acids in their sequences, especially in the catalytic active site (KXD(X)_n_DD) [13]. In human telomerase, conserved catalytic active site residues are Asp712, Asp868, Asp869 and Lys710 [13]. The catalytic active site in *T. castaneum* telomerase has three conserved aspartic acid residues (Asp343, Asp344, Asp251) which coordinate to Mg^2+^, and Lys372 provides the base for the deoxynucleotide condensation reaction [14,15]. Focused molecular docking results showed that telomerase is inhibited by boldine and its derivative BSB with inhibition constants of 9.15 μM and 221.08 nM, respectively (Table 1). The numbering of atoms in the protein and ligands reported in text and figures are all based on the software outputs. In the best binding pose of boldine, Energy of Binding (EB) = −6.87 kJ/mol and inhibitory constant (Ki) = 9.15 μM, the alkaloid interacted with telomerase via three hydrogen bonds: between the backbone NH of Ala255 as donor and O2 of the ligand as acceptor, between the H atom of Asn369 as donor and O4 of the ligand, and finally between the hydrogen atom in the amide group of Lys372 as donor atom and a hydroxyl oxygen atom of the ligand. BSB binds to TERT with the best EB of −9.08 kJ/mol and the best Ki of 221.08 nM via one hydrogen bond between O1 of the ligand and the NH hydrogen atom of Ile252 of telomerase at a distance of 1.4 Å (Table 1). 

Blind docking was performed to detect the possible binding mode and sites of boldine and BSB on TERT. Blind docking results showed that both ligands may bind to TERT in a binding box different from the active site with inhibition constants of 9.20 μM and 130.61 nM, respectively (Table 1). In the new binding box, in the best binding pose, EB for boldine was −6.64 kJ/mol and inhibition constant (Ki) 13.49 μM, interacting with TERT via one hydrogen bond: the NH hydrogen atom of Arg181 as donor atom to O1 of boldine, at a distance of 1.2 Å. In the best binding pose for BSB, EB = −9.39 kJ/mol and Ki = 130.61 nM, with the ligand bound to TERT through two hydrogen bonds: a hydroxyl hydrogen atom of BSB as donor and the oxygen atom of Arg181 as acceptor at a distance of 2.2 Å, and a hydrogen of Asn 192 and O4 of BSB at a distance of 2.5 Å. 

#### 2.3.2. Dynamics Simulation Results of Ligand-Protein Complexes

In the molecular docking position of TERT, boldine bound to the active site with a binding energy of −6.87 kcal/mol and an inhibition constant of 9.15 μM, and compound BSB did so with a binding energy of −9.08 kcal/mol and an inhibition constant of 221.08 nm. In the molecular dynamics simulation resulting from the blind docking position in TERT, boldine exhibited a binding energy of −6.64 kcal/mol and an inhibition constant of 9.15 μM. However, BSB binds much more strongly: binding energy = −9.39 kcal/mol and inhibition constant = 0.130 μM. Molecular dynamics simulations were performed to investigate the dynamics of boldine and the BSB interactions with TERT at the atomic level.

##### System Energy

**Figure 3:**
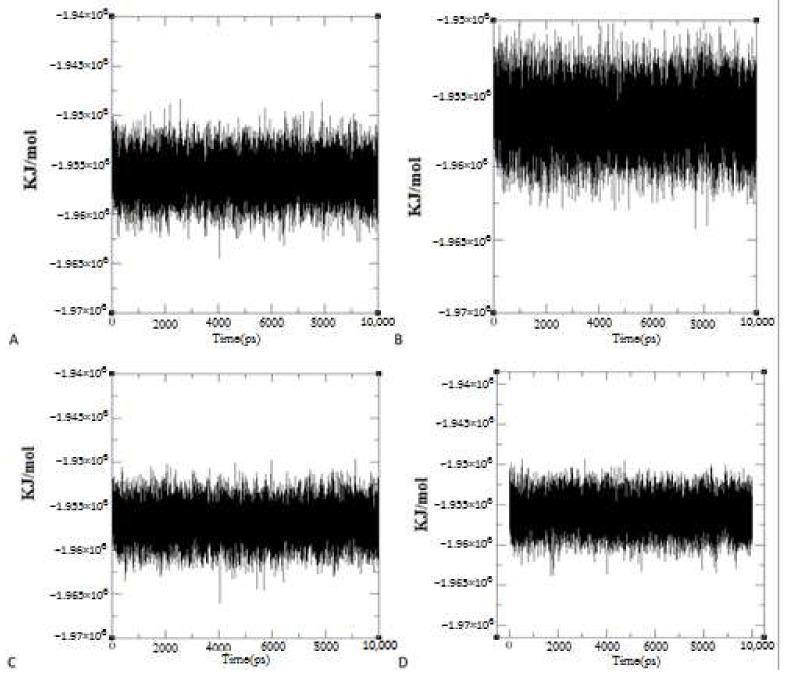
Energy of systems during 10 ns molecular dynamics simulations. Charts of Energy Changes in Protein-Ligand Complex Systems between (**A**) TERT and boldine and (**B**) TERT and BSB, during trajectories from focused docking poses in the Molecular Dynamics simulations. Charts of Energy Changes in Protein-Ligand Complex Systems between (**C**) TERT and boldine and (**D**) TERT and BSB during trajectories from blind docking poses in the Molecular Dynamics Simulations.

To investigate the stability of the whole system during the molecular dynamics simulations, the system energy for the different systems was calculated (Figure 3). Both complexes were relatively stable in terms of energy.

##### Overall Structural Stability

**Figure 4:**
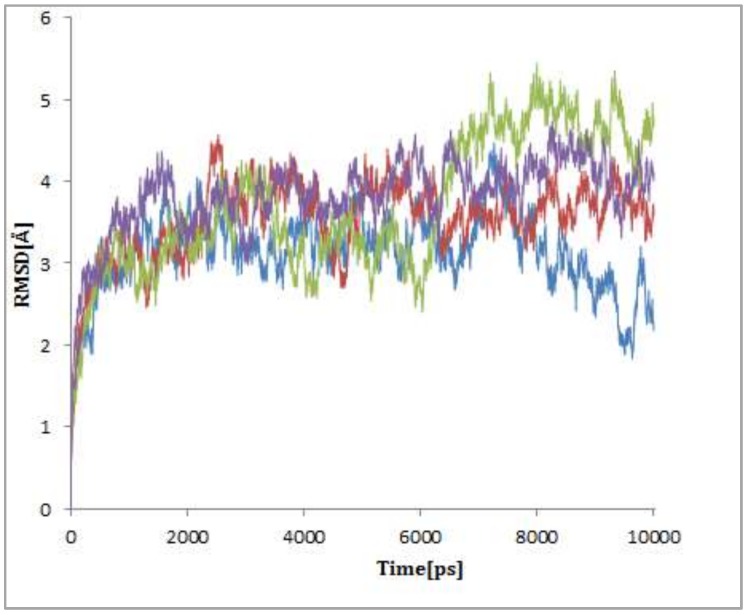
Root mean square deviations (RMSDs) of different complexes during 10 ns of molecular dynamics simulations; RMSDs of boldine (in blue and red) and BSB (green and purple) as ligands in complex with TERT after blind and focused docking screens, respectively.

The dynamic stability of boldine and BSB was studied by calculating the root mean square deviations (RMSDs) during the molecular dynamics simulations. Results of RMSDs of the model structures are shown in Figure 3. As can be seen, the simulations finally reached stable states. Detailed analyses showed that RMSD values increased rapidly for the first 0.5 ns. Then, between 0.5 and 3 ns, the RMSD values continued to increase to reach stable configurations. The RMSDs of ligand BSB starting from the initial position obtained from the blind docking screen are stable during 2 to 6 ns but they then increased suddenly to reach stable configurations (the green line in Figure 4). These sudden changes of RMSD values may reflect changes in the structure of the protein-ligand complex. It is noteworthy that the BSB structure is much more flexible than that of boldine and other derivatives that were tested and found to be relatively inactive.

The lower average of RMSDs of boldine with the initial position obtained from the blind docking screen in comparison with the binding box of boldine at the initial position obtained from the focused docking screen, during the 10 ns molecular dynamics simulations, confirmed that boldine in the new binding box located on Lys179, Pro180, Arg181, Gly182, Arg205, Asn286 and Asn290 has a more stable interaction with TERT (the blue line in Figure 4). 

**Figure 5:**
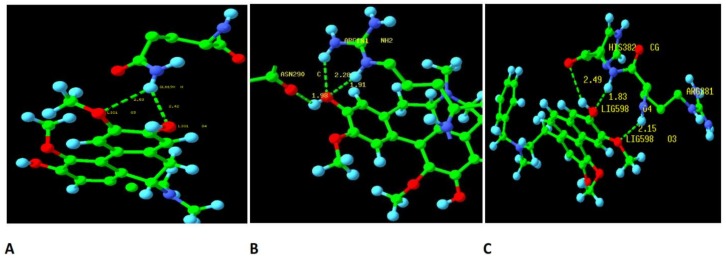
The hydrogen bond plot after 10 ns molecular dynamics simulations between (**A**) boldine (initial structure obtained after focused docking screen) in complex with TERT; (**B**) boldine (initial structure obtained after blind docking screen) in complex with TERT; (**C**) BSB (initial structure obtained after focused docking screen) in complex with TERT. The carbon, nitrogen, oxygen and hydrogen atoms were shown in green, blue, red and light blue colors, respectively.

**Figure 6:**
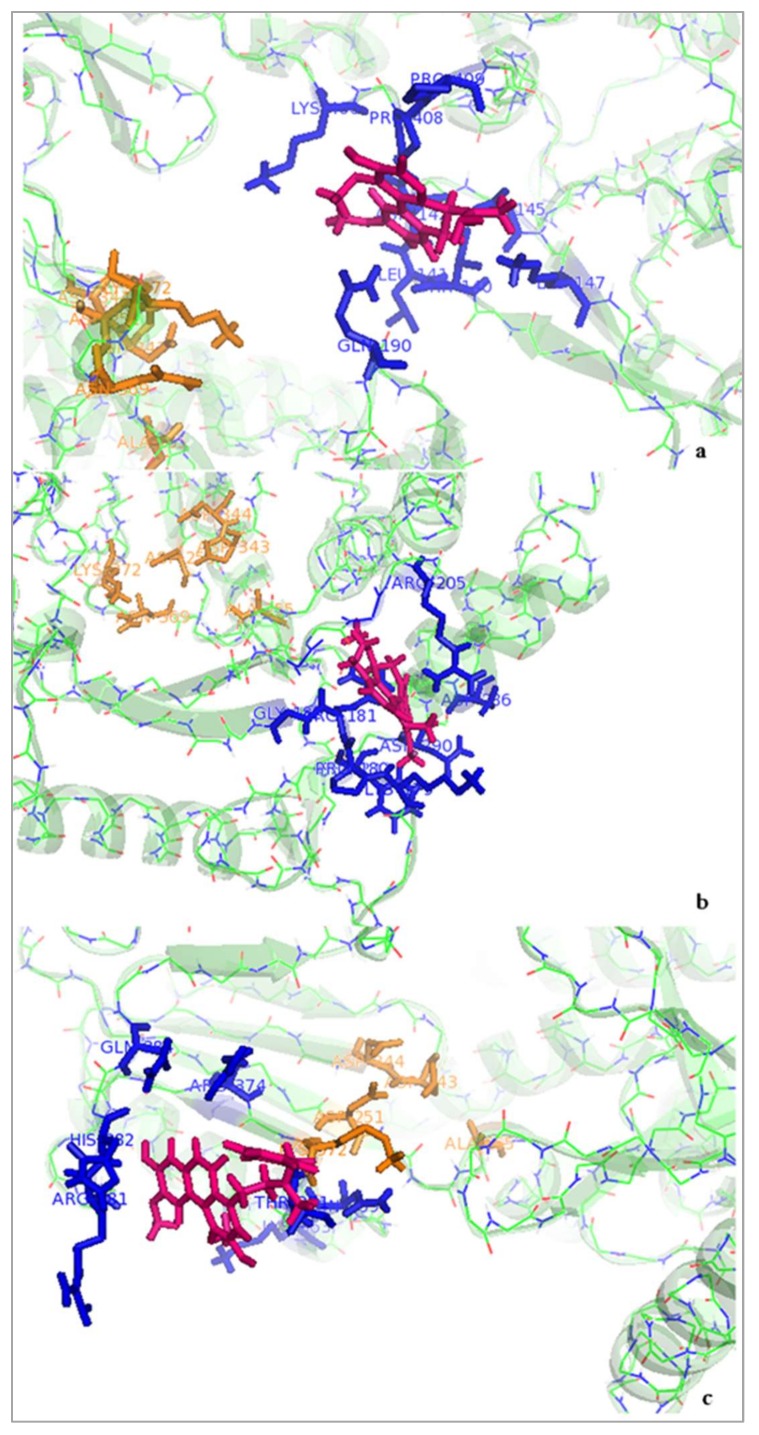
Interactions of Tc-telomerase with boldine or BSB (pink color) after 10 ns simulation; (**a**) boldine in complex with a new binding box (blue color) in finger domain of Tc-TERT that places it outside of the active site (orange color) (initial structure of boldine obtained from the focused docking screen); (**b**) boldine in complex with a new binding box (blue color) in the finger region of Tc-TERT that places it outside of the active site (orange color) (initial structure of boldine obtained from the blind docking screen); (**c**) BSB in complex with a new binding box (blue color) in the palm domain of Tc-TERT that places it outside of the active site (orange color) (initial structure of BSB obtained from the focused docking screen).

The number of hydrogen bonds between boldine and telomerase and the initial structure of boldine for the molecular dynamics simulation selected after the blind docking screen showed that the hydrogen bonding pattern of boldine with TERT during the trajectory switches between the amide hydrogen of Gln190 as donor and O3 and O4 of boldine as acceptors at distances of 2.53 and 2.42 Å, respectively. Comparing results collected from docking and the molecular dynamics simulation, it seems that boldine is released from the active site and occupies a new box on the other side of the telomerase valley where it is surrounded by Lys406, Gln190, Thr140, Asn142, Lys147 and Pro408 (Figure 5a and Figure 6a). It is concluded that the interaction between boldine and this new box is stronger than with the active site RT domain of TERT.

Also, it seems that the initial structure of boldine in complex with telomerase for the molecular dynamics simulation, selected after the blind docking screen, is more stable than the complex selected from the focused docking screen, because it connects to Arg181 through two hydrogen bonds and to As 290 with one hydrogen bond in the new binding pocket located on Lys179, Pro180, Arg181, Gly182, Arg205, Asn286 and Asn290 (Figure 5b and Figure 6b).

The molecular dynamics simulation of BSB in complex with TERT after the focused docking screen showed that the backbone NH hydrogen between Arg381 and His382 is involved in hydrogen bond interactions with O3 and O4 of the ligand at distances of 2.15 and 1.83 Å, respectively. Again, BSB releases the active site and moves to a new neighboring binding site including Lys372, Asp251, Thr371, Gln384, His382, Arg381 and Arg374 (Figure 5c and Figure 6c). Two amino acids, Asp251 and Lys372, are shared between the new binding box and the active site of TERT in complex with BSB. Also, the active site of TERT and the new binding site for BSB have similar charged residues. The increased number of hydrogen bonds established between BSB and TERT indicates that the ligand binds more strongly to the new binding box on the other side of the active site than to the active site itself.

## 3. Discussion

Boldo leaves have been used in various preparations and formulas in traditional medicine. Several beneficial effects including anti-oxidative, anti-pyretic, anti-proliferative and hepatoprotective have been recorded for boldine, erroneously assumed to be the main alkaloid of the extract. We previously found an additional mechanism for the anti-proliferative effects of boldine, based on telomerase inhibition. In the present study we investigated the strength of this effect in a synthetic derivative of boldine, *N*-benzylsecoboldine (BSB). Anti-proliferation experiments on boldine against MCF7 and MDA-MB-231 cells exhibited LD_50_ values of 160 and 150 µM after 48 h treatment, respectively. Similar experiments with BSB gave roughly 10 times lower LD_50_ values than boldine, as the most potent boldine derivative tested against breast cancer cell lines MCF7 and MDA-MB231. The greater hydrophobicity and more flexible structure of BSB in comparison with boldine and other derivatives preserving the aporphine skeleton may play critical roles in enhancing the interaction with its molecular targets inside the cell.

qTRAP-ligand assays in the presence of BSB determined its IC_50_ value as 0.17 ± 0.1 µM against *Tribolium castaneum* telomerase. Considering the IC_50_ value of 68 ± 2.5 µM for its parent natural compound, boldine, BSB showed roughly 400 times stronger direct inhibition of telomerase activity. This may be explained by stronger interactions between BSB and telomerase. It is known that boldine does not interact with its substrate, the oligonucleotide of the telomeric repeats [12]. However, the main interaction is expected to be with TERT, the catalytic subunit of telomerase.

No crystal structure of human TERT is available. Considering the highly conserved function and structure of this enzyme among species, we investigated whether boldine and the derivatives bind with the crystal structure of telomerase from *T. castaneum* (3KYL). In our focused and blind docking studies, boldine interacted well with TERT through two different binding sites: one inside the enzyme active site where it accepts the substrates and another in the vicinity of the active site. In general, interactions of the ligands with the second binding site were evaluated as moderately stronger than with the active site. However, molecular dynamics studies showed that neither boldine nor BSB reside stably in the active site but are released and attach to the second binding site, as will be explained in more detail here.

Telomerase enzymes contain two essential subunits; a catalytic protein (TERT) and an integral RNA component (TER) [15,16]. TERT is considerably diverse in size, structure and sequence between species but there are conserved secondary structures and motifs in the core of its structural elements, which in turn suggest a common mechanism of telomere replication among different species [17,18]. The TERT protein has several functional domains: TEN (Telomerase Essential N-terminal domain), TRBD (Telomerase RNA Binding Domain), RT (Reverse Transcriptase domain), and a C-Terminal Extension (CTE) [19,20,21]. Although the essential TEN domain is absent in *T. castaneum* TERT, we obtained its available 3D structure for our modeling studies [22]. The TRBD domain contains a CP motif, a QFP motif and part of the TS motif that are common to human and *T. castaneum* telomerase, but human telomerase has two additional α-helices (residues 415–456) [13]. The central catalytic RT domain including seven conserved domains has two recognizable subdomains: the “fingers” and “palm”. The “fingers” region is responsible for nucleotide binding and processing while the “palm” provides the polymerase catalytic residues with a conserved amino acid signature (KXD(X)_n_DD) in its active site and DNA primer grip. This sequence in human TERT includes the catalytic triad of Asp712, Asp868, Asp869 in addition to Lys 710, while in *T. castaneum* it is composed of Asp343, Asp344, Asp251 and lys372 [13,15,16]. The C-terminal extension interacts with DNA and has been proposed to correspond to the RT “thumb” domain [13,23].

Based on our focused docking and molecular dynamics simulations, boldine and BSB release the active site in *T. castaneum* TERT and relocate to a new box. Since there are conserved active site residues in *T. castaneum* and human TERTs, it is suggested that boldine and BSB may similarly inhibit human telomerase as non-competitive inhibitors.

Our results showed that boldine and its derivative BSB have a strong inhibitory effect on *T. castaneum* TERT. Boldine inhibits telomerase by connecting to a Gln190 amide hydrogen via two hydrogen bonds in a binding pocket surrounded by Lys406, Gln190, Thr140, Asn142, Lys147 and Pro408 and Arg181 and Asn290 in the finger domain of *T. castaneum* TERT. This location conforms to the small loop beside conserved motif 2 of the RT fingers domain. Steczkiewicz et al. show two residues, K626 and R631, in motif 2 in the RT domain while K902 of motif D in the RT palm may interact with both sugar rings and phosphate groups of the base of the telomeric DNA substrate in human TERT [13]. Residues K626 and R631 in motif 2 in the RT domain and K902 from motif D in the RT palm in human TERT correspond to K189 and R194 in motif 2 of the RT domain and K372 of motif D in the RT palm in *T. castaneum* TERT. Boldine locates at a position beside motif 2, in the finger domain of *T. castaneum* TERT, and accordingly it may also locate at the correlated site in human TERT.

In conclusion, BSB (*N*-benzylsecoboldine) strongly interacts at sub-micromolar concentrations with the catalytic subunit of *T. castaneum* TERT. It docks close to the active site in a binding pocket that is surrounded by Asp251, Thr 371, Lys 372, Arg 381, His 382, Gln 384, and Arg 374 in the palm domain of *T. castaneum* TERT. This lies in front of the site that interacts with telomeric DNA in motif 2 (K626:K189 and R631:R194) in the RT domain, with K902:K372 from motif D in the RT palm of human and *T. castaneum* TERT, respectively. As these regions are highly conserved in human and *T. castaneum* TERT, our results suggest that BSB probably interferes with the substrate-enzyme interaction as a non-competitive inhibitor.

Although data obtained from both computational and experimental studies support the inhibitory potencies of boldine and the derivative BSB on TERT, clarifying the exact details on involvement of each of the mentioned residues in ligand binding and inhibition mode requires further studies using pivotal techniques such as in vitro mutagenesis.

## 4. Materials and Methods

### 4.1. Chemicals

All chemicals except those mentioned separately were obtained at extra pure quality from Sigma-Aldrch or Merck (Germany). Boldine hydrochloride was prepared from boldine isolated from *Peumus boldus* (boldo) bark as its 1:1 complex with chloroform [24], recrystallized in 2-propanol and converted into the salt by standard procedures [25]. *N*-Benzylsecoboldine hydrochloride (BSB) was prepared from boldine free base by successive *N*-benzylation and Hofmann elimination of the obtained *N*-benzylboldinium chloride, as described in the literature [26]. Both compounds were ≥98% pure as determined by high-resolution ^1^H NMR. The chemical structure of both compounds is shown in Figure 1.

### 4.2. Cell Culture and Cytotoxicity Assay

The breast cancer cell lines MCF7 and MDA-MB-231 were grown in Dulbecco’s modified Eagle’s medium supplemented (DMEM High Glucose with stable glutamine) with 10% fetal bovine serum (FBS gold), 100 U/mL penicillin, and 100 µg/mL streptomycin in a humidified atmosphere containing 5% CO_2_ at 37 °C (all materials were purchased from PAA, Pasching, Austria) and sub-cultured routinely after reaching almost 80% confluence. Cell viability was evaluated using the trypan blue exclusion method [27]. Boldine and the derivative were dissolved in absolute ethanol (Merck, Darmstadt, Germany) at a concentration of 10 or 50 mM (stock solution) and stored at −20 °C until use. In cell treatment tests each stock solution was diluted with medium before use and the maximum final concentration of ethanol in cell cultures did not exceed 1%. A 1:2 or 1:4 serial dilution of this medium was applied for the treatments.

Cell viability was evaluated using the MTT (3-(4,5-dimethylthiazol-2-yl)-2,5-diphenyl- tetrazolium bromide) (Sigma-Aldrich, Darmstadt, Germany) assay [28]. Briefly, cells were seeded in 96-well plates at a density of 1 × 10^4^ cells/well and treated with each compound at serially diluted concentrations. After various incubation times, MTT at 0.5 mg/mL final concentration was added and incubated for 4 h to be reduced to formazan by viable cells. The concentration of this blue dye, after dissolving in DMSO containing 10% SDS and 1% acetic acid, was measured by absorbance measurement at 570 nm using a plate reader (BioTek, Winooski, VT, USA), and then cell viability was calculated using Gen5 software version 1.06. The assay was carried out at least in three independent logical repeats, each of which included samples in triplicates. The concentration of boldine and the derivatives that caused cell growth to decrease to 50% of untreated controls, IC_50_, was determined from the dose–response curves. The results were analyzed using one way ANOVA with post-hoc Tukey HSD test and reported as means ± SEM. 

### 4.3. q-TRAP-Ligand Assay

Direct treatment of telomerase in cell lysates with boldine and the derivatives was performed using the q-TRAP-ligand assay as described earlier [12]. Briefly, sub-confluent MCF7 cells were collected, washed with PBS, lysed in an ice-cold buffer containing 10 mM Tris-HCl pH =7 .5, 1 mM MgCl_2_, 1 mM EGTA, 0.1 mM phenylmethylsulfonylfluoride (PMSF), 5 mM beta-mercaptoethanol, 0.5% CHAPS and 10% glycerol and centrifuged at 14,000× *g*. The protein concentration of the collected supernatant was measured using the microBradford assay. Q-TRAP-ligand assay is based on a SYBR-Green quantitative telomere repeat amplification protocol (q-TRAP) [29] with some small modifications [12,30]. In general, the assay depends on two enzymes: telomerase which elongates the synthetic substrate, TS, and hot-start Taq polymerase for PCR amplification. Therefore, in q-TRAP-ligand experiments (also known as the in vitro TRAP assay), the reaction mixtures were treated with the desired compound in two distinct steps to differentiate between its potential effects on the enzymes. Briefly, a master mix of q-TRAP reaction including 0.5 µg total protein of MCF-7 cell lysate, 1× SYBR Green Master Mix (GenetBio, Chungcheonam-Do, Korea), and 10 pmol TS (5′-AATCCGTCGAGCAGATT-3′) was prepared and aliquoted to two sets on ice. Samples in each set were treated with different final concentrations of the compound BSB (0, 0.005, 0.05, 0.5 and 5 µM), boldine (0, 5, 10, 50, 100 and 150 µM) at the specified step, before adding the telomerase substrate (T) and before the amplification step (P), both with 30 min incubation on ice. In set T, the compound was added before telomerase activity, which means that both telomerase and Taq polymerase were exposed to the boldine or the derivative. However, in set P, only hot-start Taq was affected. All samples were incubated for 20 min at 25 °C to extend the TS primer by telomerase. Both sets were put back on ice and then the compound was added to set P and after 30 min the amplification step was performed. Five pmol ACX (5′-GCGCGGCTTACCCTTACCCTTACCCTAACC-3′) primer was added to all samples and the amplification of telomerase products was started at 94 °C for 10 min and 40 cycles of 30 s each at 94 °C, 30 s at 50 °C and 45 s at 72 °C with signal acquisition in a real-time thermal cycler Rotor-Gene 3000 (Corbett Research, Sydney, Australia). The threshold cycle value (*C*_t_) determined for each sample by using Rotor-Gene 6.01 software (https://www.qiagen.com/us/resources/) was compared with those of the standards generated from serially diluted cell lysates of the untreated MCF7 control. This experiment was performed at least three times with each repeat, including triplicate samples for each concentration of the desired compound. The probable traces of RNase contamination which potentially might give rise to false positive results were checked by incubating total RNA with aliquots of boldine or BSB for 30 min at room temperature followed by electrophoresis in agarose gel.

### 4.4. Computational Studies

#### 4.4.1. Protein and Ligand Preparation

The ribonucleoprotein telomerase is composed of a template containing RNA and a catalytic subunit TERT. The catalytic subunit of telomerase structures from different species collectively contain conserved components including two very important domains: an RNA binding domain (TRBD) and a reverse transcriptase domain (RT). The amino acid sequence identities between human telomerase domains TRBD and RT and the corresponding structures in the Protein Data Bank (PDB) are 22 and 24%, respectively [13]. No 3D structure of human telomerase is available, however. Among the X-ray structures of telomerases in the Protein Data Bank, one of them is the full length *Tribolium castaneum* telomerase (containing TRBD and RT domains) alone [14] and the other has the enzyme in complex with an RNA∶DNA hairpin [31]. Here, the crystal structure of *T. castaneum* telomerase (PDB code: 3KYL) was taken from the Protein Data Bank (http://www.rcsb.org/pdb) and utilized for docking runs in complex with boldine and its derivative. To prepare the proteins for docking, all non-protein related molecules including water were deleted and hydrogen atoms were added using Autodock tools 4.2 (ADT) [32].

To optimize the Tc-telomerase structure, 10 ns MD simulations were carried out with the GROMACS 4.6.5 package. The GROMOS96 force field [33,34] was applied for MD calculations. The complexes were surrounded by a cubic periodic box of SPC water molecules with 1.0 nm (10.0 Å) edges along each dimension. Sodium and chloride ions were added to the system to maintain charge neutrality. All covalent bonds to hydrogen atoms were constrained using the LINCS algorithm [35]. Electrostatic potentials were calculated using the Particle-Mesh Ewald (PME) algorithm [36] with a cutoff of 10 Å for Lennard-Jones interactions. MD integrator [37,38] was used in order to integrate the equations of motion. Periodic boundary conditions were applied to avoid edge effects. Prior to MD production, 1000 steps of steepest-descent minimization and 1500 steps of conjugate gradient minimization were applied to the entire model system. The whole system was heated to 300 K over 300 ps using the NVT (constant volume and normal temperature) ensemble with the V-rescale thermostat protocol [39]; then, 300 ps equilibrations were carried out in the NPT (constant normal pressure and normal temperature) ensemble with the Berendsen thermostat protocol [40,41]. The coordinates of all atoms in the system were saved every 1 ps during the entire MD simulation. In order to get the optimized and stable geometry of ligands, the ligand structures were optimized by Hyperchem software using the Polak-Ribière algorithm and the AMBER force field [42]. Lowest energy structures were chosen as initial geometries for docking studies.

#### 4.4.2. Molecular Docking

Docking calculation was performed using Autodock Tools 4.2 software with autodock4 and autogrid4 programs [32,43]. The files were subsequently converted to the pdbqt format to perform molecular docking using the Genetic Algorithm with Local Searching. An auto grid box was constructed in such a way that the ligand could freely move in the corresponding space (coordinates of the three dimensions [grid center]: X: 65.625, Y54.752 and Z: 44.279 and number of grid points in the three dimensions [npts]: X: 70, Y: 70 and Z: 70 for focused docking box; spacing: 0.375 and X = 126, Y = 126, Z = 126 for blind docking box). This grid box was built to encompass isoform active sites. The default docking parameters were accepted except for the maximum number of energy evaluations [ga_num_evals] and the number of runs [ga_run] that were set to 25,000,000 and 250, respectively. Docking results were visualized by AutoDock Tools [31] and PyMOL [44].

#### 4.4.3. Molecular Dynamics Simulation

The ligands, i.e., boldine and/or its derivative docked with telomerase, were used to perform explicit solvent MD simulations. MD simulations were carried out with the GROMACS 4.6.5 package (ftp://ftp.gromacs.org/pub/gromacs/gromacs-5.0.4.tar.gz) as mentioned above in Section 4.4.1. 

## Figures and Tables

**Table 1:** Results of focused and blind docking of boldine and *N*-Benzylsecoboldine (BSB) with telomerase. (HB: hydrogen bonding; EB: energy of binding; Ki: Inhibitory constant).

	Focused Docking		Blind Docking	
	Boldine	BSB	Boldine	BSB
**Ki (μM)**	9.15	0.22108	9.15	0.130
**Binding Energy (kJ/mol)**	−6.87	−9.08	−6.64	−9.39
**Number of Hydrogen Bonds**	3	1	1	2
**Amino Acids**	Ala255 Asn369 Lys372	Ile252	Arg181	Arg181 Pro180
**RMSD (Å)**	84.54	81.53	72.13	73.82

## References

[1] O’Brien P., Carrasco-Pozo C., Speisky H. (2006). Boldine and its Antioxidant or Health-Promoting Properties. Chem.-Biol. Interact..

[2] Han Z., Zheng Y., Chen N., Luan L., Zhou C., Gan L., Wu Y. (2008). Simultaneous Determination of Four Alkaloids in *Lindera aggregata* by Ultra-High-Pressure Liquid Chromatography–Tandem Mass Spectrometry. J. Chromatogr. A.

[3] Zhang A., Zhang Y., Branfman A.R., Baldessarini R.J., Neumeyer J.L. (2007). Advances in Development of Dopaminergic Aporphinoids. J. Med. Chem..

[4] Walstab J., Wohlfarth C., Hovius R., Schmitteckert S., Röth R., Lasitschka F., Wink M., Bönisch H., Niesler B. (2014). Natural compounds boldine and menthol are antagonists of human 5-HT_3_ receptors: Implications for treating gastrointestinal disorders. Neurogastroenterol. Motil..

[5] Lau Y.S., Ling W.C., Murugan D., Mustafa M.R. (2015). Boldine Ameliorates Vascular Oxidative Stress and Endothelial Dysfunction: Therapeutic Implication for Hypertension and Diabetes. J. Cardiovasc. Pharmacol..

[6] Hernández-Salinas R., Vielma A.Z., Arismendi M.N., Boric M.P., Sáez J.C., Velarde V. (2013). Boldine prevents renal alterations in diabetic rats. J. Diabetes Res..

[7] Lau Y.S., Tian X.Y., Mustafa M.R., Murugan D., Liu J., Zhang Y., Lau C.W., Huang Y. (2013). Boldine improves endothelial function in diabetic db/db mice through inhibition of angiotensin II-mediated BMP4-oxidative stress cascade. Br. J. Pharmacol..

[8] Gerhardt D., Bertola G., Dietrich F., Figueiró F., Zanotto-Filho A., Fonseca J.C.M., Morrone F.B., Barrios C.H., Battastini A.M.O., Salbego C.G. (2014). Salbego Boldine induces cell cycle arrest and apoptosis in T24 human bladder cancer cell line via regulation of ERK, AKT, and GSK-3β. Urol. Oncol. Semin. Orig. Investig..

[9] Tomšík P., Mičuda S., Muthná D., Čermáková E., Havelek R., Rudolf E., Hroch M., Kadová Z., Řezáčová M., Ćmielová J. (2016). Boldine Inhibits Mouse Mammary Carcinoma in vivo and Human MCF-7 Breast Cancer Cells in vitro. Planta Med..

[10] Qiu X., Shi L., Zhuang H., Zhang H., Wang J., Wang L., Sun P., Yu L., Liu L. (2017). Cerebrovascular Protective Effect of Boldine Against Neural Apoptosis via Inhibition of Mitochondrial Bax Translocation and Cytochrome C Release. Med. Sci. Monit..

[11] Noureini S.K., Wink M. (2015). Dose-dependent cytotoxic effects of boldine in HepG-2 cells-telomerase inhibition and apoptosis induction. Molecules.

[12] Kazemi Noureini S., Tanavar F. (2015). Boldine, a natural aporphine alkaloid, inhibits telomerase at non-toxic concentrations. Chem. Biol. Interact..

[13] Steczkiewicz K., Zimmermann M.T., Kurcinski M., Lewis B.A., Dobbs D., Kloczkowski A., Jernigan R.L., Kolinski A., Ginalski K. (2011). Human telomerase model shows the role of the TEN domain in advancing the double helix for the next polymerization step. Proc. Natl. Acad. Sci. USA.

[14] Gillis A.J., Schuller A.P., Skordalakes E. (2008). Structure of the *Tribolium castaneum* telomerase catalytic subunit TERT. Nature.

[15] Feng J., Funk D.W., Wang S.S., Weinrich S.L., Avilion A.A., Chiu P.C., Adams R.R., Chang E., Allsopp R.C., Yu J. (1995). The RNA component of human telomerase. Science.

[16] Nakamura T.M., Morin G.B., Chapman K.B., Weinrich S.L., Andrews W.H., Lingner J., Harley C.B., Cech T.R. (1997). Telomerase catalytic subunit homologs from fission yeast and human. Science.

[17] Chen J.L., Greider C.W. (2004). An emerging consensus for telomerase RNA structure. Proc. Natl. Acad. Sci. USA.

[18] Ly H., Blackburn E.H., Parslow T.G. (2003). Comprehensive structure–function analysis of the core domain of human telomerase RNA. Mol. Cell. Biol..

[19] Autexier C., Lue N.F. (2006). The structure and function of telomerase reverse transcriptase. Annu. Rev. Biochem..

[20] Wyatt H.D., West S.C., Beattie T.L. (2010). In*TERT*preting telomerase structure and function. Nucleic. Acids Res..

[21] Sekaran V.G., Soares J., Jarstfer M.B. (2010). Structures of telomerase subunits provide functional insights. Biochim. Biophys. Acta.

[22] Steitz T.A. (1997). DNA and RNA polymerases: Structural diversity and common mechanisms. Harvey Lect..

[23] Hossain S., Singh S., Lue N.F. (2002). Functional analysis of the C-terminal extension of telomerase reverse transcriptase. A putative “thumb” domain. J. Biol. Chem..

[24] Asencio M., Cassels B.K., Manríquez V., Boys D. (1996). (*S*)-1,10-Dimethoxy-2,9-dihydroxyaporphinium chloride (boldine hydrochloride), C_19_H_22_NO_4_Cl. Acta Crystallogr..

[25] Teng C.M., Yu S.M., Pan C.P., Lee S.S. (1994). Mechanism of vasorelaxation caused by IM-benzylsecoboldine in rat thoracic aorta. J. Biomed. Sci..

[26] Sobarzo-Sánchez E., Cassels B.K., Saitz-Barría C., Jullian C. (2001). Oxazine- and oxazole-fused derivatives of the alkaloid boldine and their complete structural and spectral assignment by HMQC and HMBC experiments. Magn. Reson. Chem..

[27] Majno G., Joris I. (1995). Apoptosis, oncosis, and necrosis. An overview of cell death. Am. J. Pathol..

[28] Mosmann T. (1983). Rapid colorimetric assay for cellular growth and survival: Application to proliferation and cytotoxicity assays. J. Immunol. Methods.

[29] Hou M., Xu D., Björkholm M., Gruber A. (2001). Real-time quantitative telomeric repeat amplification protocol assay for the detection of telomerase activity. Clin. Chem..

[30] Kazemi Noureini S., Wink M. (2012). Antiproliferative Effects of Crocin in HepG2 Cells by Telomerase Inhibition and hTERT Down-Regulation. Asian Pac. J. Cancer Prev..

[31] Mitchell M., Gillis A., Futahashi M., Fujiwara H., Skordalakes E. (2010). Structural basis for telomerase catalytic subunit TERT binding to RNA template and telomeric DNA. Nat. Struct. Mol. Biol..

[32] Morris G.M., Huey R., Lindstrom W., Sanner M.F., Belew R.K., Goodsell D.S., Olson A.J. (2009). AutoDock4 and AutoDockTools4: Automated Docking with Selective Receptor Flexibility. J. Comput. Chem..

[33] Lemkul J.A., Allen W.J., Bevan D.R. (2010). Practical Considerations for Building GROMOS-Compatible Small-Molecule Topologies. J. Chem. Inf. Model..

[34] Oostenbrink C., Villa A., Mark A.E., Gunsteren W.F.V. (2004). A Biomolecular Force Field Based on the Free Enthalpy of Hydration and Solvation: The GROMOS Force-Field Parameter Sets 53A5 and 53A6. J. Comput. Chem..

[35] Ryckaert J.P., Ciccotti G., Berendsen H.J.C. (1977). Numerical integrationof the Cartesian equations of motion of a system with constraints: Molecular dynamics of n-alkanes. J. Comput. Phys..

[36] Darden T., York D., Pedersen L. (1993). Particle mesh Ewald: An Nlog (N) method for Ewald sums in large systems. J. Chem. Phys..

[37] Tuckerman M., Berne B.J., Martyna G.J. (1992). Reversible multiple time scale molecular dynamics. J. Chem. Phys..

[38] Bou-Rabee N. (2014). Time Integrators for Molecular Dynamics. Entropy.

[39] Wong-ekkabut J., Karttunen M. (2012). Assessment of Common Simulation Protocols for Simulations of Nanopores, Membrane Proteins, and Channels. J. Chem. Theory Comput..

[40] Alvarez H.A., McCarthy A.N., Grigera J.R. (2012). A Molecular Dynamics Approach to Ligand-Receptor Interactionin the Aspirin-Human Serum Albumin Complex. Biophys. J..

[41] Prathab B., Aminabhavi T.M. (2007). Molecular Modeling Study on Surface, Thermal, Mechanical and Gas Diffusion Properties of Chitosan. Polym. Phys..

[42] Schuttelkopf A.W., van Aalten D.M.F. (2004). PRODRG: A tool for high-throughput crystallography of protein-ligand complexes. Acta Crystallogr..

[43] Maunz A., Gütlein M., Rautenberg M., Vorgrimmler D., Gebele D., Helma C. (2013). Lazar: A modular predictive toxicology framework. Front. Pharmacol..

[44] DeLano W.L. (2002). The PyMOL User’s Manual.

